# Morphological variation and expressed sequence tags-simple sequence repeats-based genetic diversity of *Aspergillus cristatus* in Chinese dark tea

**DOI:** 10.3389/fmicb.2024.1390030

**Published:** 2024-06-03

**Authors:** Zhiyuan Hu, Shiquan Liu, Xiaohong Zhou, Zhanjun Liu, Taotao Li, Songlin Yu, Xinyu Zhang, Zhenggang Xu

**Affiliations:** ^1^Hunan Provincial Key Lab of Dark Tea and Jin-hua, School of Materials and Chemical Engineering, Hunan City University, Yiyang, China; ^2^College of Forestry, Northwest A & F University, Yangling, China; ^3^Research Institute of South Tea Introduced to North in Huashan, Weinan, China

**Keywords:** *Aspergillus cristatus*, molecular marker, cluster analysis, polymorphism, microbial resources

## Abstract

**Introduction:**

*Aspergillus cristatus* is a homothallic fungus that is used in the natural fermentation process of Chinese Fuzhuan tea and has been linked to the production of bioactive components. However, not much is known about the variations present in the fungus. To understand the variation of the dominant microorganism, *A. cristatus*, within dark tea, the present study investigated the genetic and morphological diversity of 70 *A. cristatus* collected across six provinces of China.

**Methods:**

Expressed sequence tags-simple sequence repeats (EST-SSR) loci for *A. cristatus* were identified and corresponding primers were developed. Subsequently, 15 specimens were selected for PCR amplification.

**Results:**

The phylogenetic tree obtained revealed four distinct clusters with a genetic similarity coefficient of 0.983, corresponding to previously identified morphological groups. Five strains (A1, A11, B1, D1, and JH1805) with considerable differences in EST-SSR results were selected for further physiological variation investigation. Microstructural examinations revealed no apparent differentiation among the representative strains. However, colony morphology under a range of culture media varied substantially between strains, as did the extracellular enzymatic activity (cellulase, pectinase, protease, and polyphenol oxidase); the data indicate that there are differences in physiological metabolic capacity among *A. cristatus* strains.

**Discussion:**

Notably, JH1805, B1, and A11 exhibited higher enzymatic activity, indicating their potential application in the production of genetically improved strains. The findings provide valuable insights into species identification, genetic diversity determination, and marker-assisted breeding strategies for *A. cristatus*.

## 1 Introduction

*Aspergillus cristatus*, known as the “Golden flower fungus,” is the sexual type of *Eurotium cristatum* (Xiao et al., 2020; Efimenko et al., 2021), and it is the predominant microorganism in Fuzhuan tea, which is a notable dark tea variant produced through the fermentation of *Camellia sinensis* (An et al., 2021; Zhang et al., 2022a). The fungus ferments tea leaves and secretes enzymes that catalyze the oxidation, polymerization, transformation, and degradation of cellulose, pectin, fat, and polyphenols. The enzymatic activity reduces undesirable bitterness, while contributing to the distinctive color, aroma, and taste of dark tea (Xiao et al., 2021; Du et al., 2022; Xiao et al., 2022). Traditionally, *A. cristatus* was used for the processing of Fuzhuan tea. However, advances in production technology in recent years have enabled the development of different types of dark tea products, such as “loose dark” (Yao et al., 2017; Chen et al., 2023), “instant dark” (An et al., 2021; Chen et al., 2021), and “Pu′er” teas (Jiang et al., 2018) using *A. cristatus*, which effectively improved the quality of the tea. Additionally, *A. cristatus* fermentation yields a range of bioactive components, including monacolin K, which regulates blood lipids (Lu et al., 2022a), antibacterial benzaldehyde derivatives (Shi et al., 2019), amide derivatives (Wen et al., 2022), flavonoids with hypoglycemic effects (Zhao et al., 2023a), and fungal polysaccharides that modulate intestinal flora composition (Lu et al., 2022b). The health benefits of dark tea have made it highly desirable among consumers. In 2022, China’s dark tea output reached 426,300 tons, an increase of approximately 148% compared with 172,000 tons in 2012. Recently, researchers have identified the potential use of *A. cristatus* to improving the quality of other plant materials such as mulberry leaves (Yang et al., 2023), *Ginkgo biloba* seeds (Zou et al., 2022), turmeric (Xiang et al., 2020), and *Siraitia grosvenorii* (Yin et al., 2023a). Beyond catering to the health needs of contemporary consumers, *A. cristatus*-fermented foods also offer opportunities for deep processing and diversified utilization of agricultural and forestry products.

As a crucial microbial resource, *A. cristatus* has been extensively investigated with regard to fermentation technology, metabolites, and healthcare effects; however, its genetic diversity remains poorly understood. Because dark tea production is widely distributed in China, variations in geography and anthropogenic factors likely contribute to *A. cristatus* evolution, as some researchers have discovered that *A. cristatus* isolated from different dark teas does not form complete homogenous populations regarding their physical characteristics. Furthermore, previous studies have provided supporting evidence that confirms the presence of diversity within *A. cristatus*. Li et al. (2023a) conducted a study on 20 wild strains of *A. cristatus*, which exhibited diverse morphological traits, and they determined that only six were capable of lovastatin (a lipid-lowering drug) biosynthesis. Moreover, the observed yields metabolized varied substantially. Wang et al. (2019) compared the internal transcribed spacer (ITS) sequence differences of 33 *A. cristatus* and found that 24 were different from that of the standard strain to varying degrees (>99%); in addition, different strains had certain variations in their morphology and growth rate. Shi et al. (2019) analyzed benzaldehyde derivatives from eight *A. cristatus* using the high-performance liquid chromatography, revealing substantial variations in both the composition and yield of metabolic benzaldehyde derivatives among the different strains. Furthermore, Wang et al. (2022a) determined that the ITS sequences of 18 *A. cristatus* strains isolated from Fuzhuan tea demonstrated high similarity (with one exception showing 98.46% similarity to corresponding reference strains, and the rest being ≥99%), with notable differences among the strains in terms of tolerance to acidic environments, concentrations of bile salts, capacity to inhibit intestinal pathogenic bacteria, as well as hydrophobicity. Because the differences influence dark tea quality, the fermentation processes must be controlled to improve the product. Such a level of control requires the elucidation of *A. cristatus* genetic diversity and strain variations. However, existing studies have some limitations. Firstly, the physiological characteristics of fungi are influenced by cultural factors, leading to inconsistent gene expression and unstable morphological identification results. Secondly, the molecular marker sequences used (such as ITS, 18S rDNA, etc.) are relatively conservative and provide less phylogenetic information, making it difficult to distinguish intraspecific units. Therefore, there is still a need for more accurate and high-resolution molecular markers to establish a basis for identifying the genetic diversity of *A. cristatus*. Expressed sequence tags-simple sequence repeats (EST-SSR) are localized within the transcriptional regions of the genome and potentially play roles similar to those of functional genes, making such markers ideal for evaluating intraspecific genetic diversity (Lu et al., 2021). Moreover, EST-SSR technology has been employed extensively in microbial population genetics, germplasm resource evaluation, and taxonomic studies (Zhang et al., 2022c; Darshan et al., 2023; Wang et al., 2023a).

In the present study, *A. cristatus* strains from different regions of China were collected to analyze their morphology, enzyme activity, and microsatellite-based genetic diversity. The aim of the study was to clarify the *A. cristatus* strains and identify candidates suitable for improving dark-tea production.

## 2 Materials and methods

### 2.1 Strain collection and culture

#### 2.1.1 Strains and culture media

**Figure 1:**
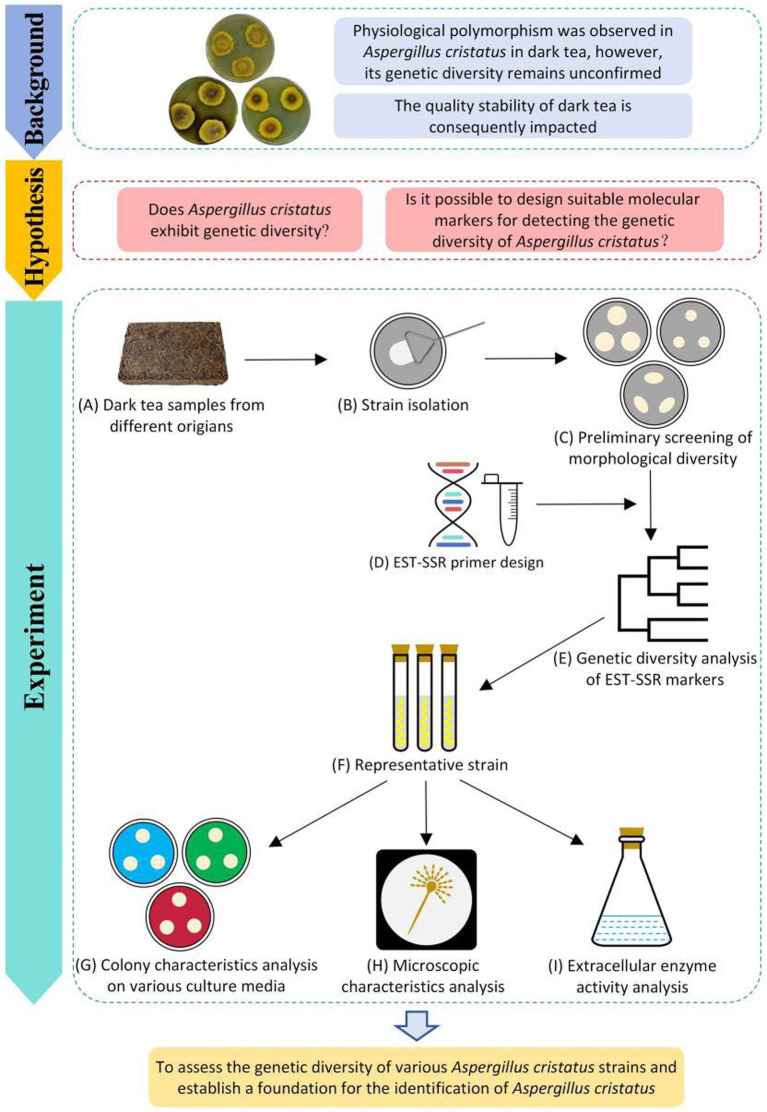
Procedures for genetic diversity analysis of *A. cristatus*.

Sixty-nine *A. cristatus* strains from dark tea samples were collected across various regions of China (Hunan, Hubei, Zhejiang, Shaanxi, Guangxi, and Guizhou Province) and isolated using a gradient dilution (Figures 1A,B). Three standard strains provided by Hunan City University (*A. cristatus* JH1805, *A. pseudoglaucus* HL1801, and *A. chevalieri* XW1803) were also included as controls for the isolated strains to effectively analyze the variation of wild-type strains. The sources of the experimental strains are listed in Supplementary Table S1.

Potato dextrose agar (PDA), modified PDA, 20% Czapek-Dox medium (20% CDA), 60% CDA, czapek yeast extract agar (CYA), potato glucose liquid medium (PDL), and dark tea liquid medium (DTL) were used as culture media. Among them, PDA, modified PDA, 20% CDA, and 60% CDA were used for morphological observations. Whereas PDL and DTL were used for the cultivation of mycelium for DNA extraction and detection of extracellular enzyme activity of the strains, respectively. The formulas for the different culture media are shown in Supplementary Table S2.

#### 2.1.2 Morphological diversity analysis of different *A. cristatus* strains

Strains were inoculated onto PDA plates and cultured at 28°C for 6 d (Figure 1C). Their characteristics were observed and recorded (for methodology, see Supplementary Figure S1). The assessment and clustering of the morphological characteristics of the colony were performed according to the method described by Yang and Song (2014). The average colony size was determined based on the crossover method. Edge shape was assessed based on the orderliness of colony edges, pigment secretion was evaluated based on the sizes and depths of melanin-containing areas in the colony center; surface characteristics were evaluated to determine colony smoothness; cluster analysis was determined using SPSS AU (Xu et al., 2023a) based on four physiological traits of the tested strains.

### 2.2 EST-SSR primer design and amplification

#### 2.2.1 Extraction, library construction, and sequencing of *A. cristatus* RNA

The RNA was extracted from sexual and asexual mycelia of *A. cristatus* JH1805 cultured on PDA medium for 6 d (Hu et al., 2023). The extracted RNA was then used for library construction and sequencing using the methods described by Hu et al. (2023).

#### 2.2.2 Screening of EST-SSR loci screening and primer design of *A. cristatus*

MISA software (Liu et al., 2023a; https://webblast.ipk-gatersleben.de/misa/) was used to identify SSR loci within the *A. cristatus* transcriptome (Figure 1D). The criteria were as follows: single nucleotide repeats >10; dinucleotide repeats >5; trinucleotide repeats >4; and tetranucleotide, pentanucleotide, and hexanucleotide repeats >3.

Primers for SSR-containing sequences ≥12 base pairs (bp) were designed in Primer Premier 5.0. The design criteria were as follows: (1) flanking sequences ≥50 bp; (2) annealing temperature (Tm) of 58–65°C, and a ≤2°C difference between the Tm of positive and negative primers; (3) expected amplicon size, 80–250 bp; (4) primer length, 18–28 bp; and (5) GC content, 40–60%. Hairpins, dimers, false priming, and cross-dimer formation were avoided as much as possible during the primer design. Primer verification was done using BLAST on the unigene library, which resulted in 30 pairs selected for subsequent primer synthesis (Supplementary Table S3).

#### 2.2.3 Amplification of EST-SSR primers in different *A. cristatus* strains

Representative strains, such as JH1805, A1, A11, A13, A12, A27, A30, B1, B8, C5, C6, D1, E1, E13, and F3 were selected from different provinces as test samples for DNA extraction (Alcaide et al., 2019; Xu et al., 2023b), and *A. chevalieri* XW1803 was used as the control. The 16 strains were cultured at 28°C in PDL liquid medium for 6 d, then centrifuged at 2550 × g for 5 min. Subsequently, mycelia were collected for DNA extraction.

Extracted DNA was amplified with the 30 EST-SSR primer pairs. The reaction mix comprised 34 μL T3 Super PCR Mix, 2 μL each of upstream and downstream primer, and 2 μL of the DNA template. The parameter settings for PCR amplification of EST-SSR sequences are shown in Supplementary Table S4. Subsequently, PCR products were electrophoresed for 120 min on a 10% polyacrylamide gel at 100 V. Bands were visualized using an EB-stained gel imaging system (Vidya et al., 2021).

#### 2.2.4 Genetic diversity of EST-SSR

To assess the suitability (presence) of SSR markers, their degree of polymorphism (based on length) was used (Zhao et al., 2023b). High, intermediate, and low polymorphism is typically defined as SSR length ≥ 20 bp, 12–19 bp, and <12 bp, respectively (Zhang et al., 2022b).

The analysis of EST-SSR genetic diversity was conducted as follows: the images captured by the gel imaging system in step 2.2.3 were analyzed, and each pair of EST-SSR primer corresponds to a locus. If a polymorphic band is observed, it is considered an allelic variation. The electrophoretic bands were analyzed statistically using the “0,1” system, where no bands were recorded as “0” and bands were recorded as “1.” The statistical results were summarized in an MS Excel table to establish the original database (Li et al., 2023b), which was used for the comparison and clustering analysis of EST-SSR amplified bands. The genetic similarity coefficients among 16 strains were analyzed using NTSYS-pc 2.2, and unweighted pair group method with arithmetic mean (UPGMA) hierarchical clustering analysis was performed based on the similarity coefficient to construct a system tree. The positions of different strains on the system tree can be used to evaluate their EST-SSR genetic diversity (Figure 1E) (Chen et al., 2022). In addition, the conjugate values and matrix correlation coefficients of the UPGMA clustering results were calculated using NTSYS-pc 2.2 to evaluate the clustering results (Yildiz et al., 2021).

The 15 *A. cristatus* phenotypic traits obtained from the PDA culture were standardized in NTSYS-pc 2.2 to obtain Euclidean distance and genetic distance matrices of EST-SSR markers. The two matrices were subjected to a Mantel correlation analysis (Yang et al., 2022).

### 2.3 Physiological characteristics of representative strains

According to the results of EST-SSR genetic diversity analysis, *A. cristatus* A1, A11, B1, and D1 were selected as representative strains from different branches of the clustering tree (Figure 1F), which was then compared with the standard strain *A. cristatus* JH1805, *A. pseudoglaucus* HL1801, and *A. chevalieri* XW1803.

#### 2.3.1 Colony characteristics analysis on various culture media

Using the three-point method, strains were inoculated onto plates containing PDA, modified PDA, 20% CDA, 60% CDA, and CYA (Figure 1G). Since culture temperature and osmotic pressure affect the reproductive pattern of *A. cristatus* (Tan et al., 2018; Shao et al., 2022), to observe the different reproductive structures of *A. cristatus*, all plates were incubated at 28°C except for 60% CDA, which was incubated at 32°C. After culturing for 6 d, colony morphology was recorded by photographing.

#### 2.3.2 Microscopic characteristics analysis

Sexual and asexual mycelia cultured in 60% CDA medium for 6 d were selected and prepared as fixed specimens (Figure 1H). Mycelium structure was observed under a scanning electron microscope (SEM). Different regions from the electron microscope images were selected for length and diameter measurements in ImageJ Software (Wainaina and Taherzadeh, 2023). Twenty samples per structure were measured randomly.

#### 2.3.3 Extracellular enzyme activity analysis

Spore suspensions (1.0 mL) for each strain were pipetted into a dark tea liquid medium and then cultivated at 28°C (Figure 1I). After fermentation for 3–8 d, suspensions were centrifuged at 8263 × g for 10 min to obtain the supernatant crude enzyme solution. The enzyme activity per unit volume was calculated by adding the volume of the sample (U/mL).

Cellulase activity was measured using the dinitrosalicylic acid (DNS) method (Sudeep et al., 2020): 0.5 mL of crude enzyme solution was added to 2.0 mL of 1.0% CMC-Na solution, and the solution was placed in a water bath at 40°C for 30 min. Subsequently, 2.0 mL of DNS solution was added to terminate the reaction immediately. The solution was then heated in boiling water for 5 min, after which it was removed and cooled. The volume was maintained at 20 mL. The optical density value was measured at 540 nm using an ultraviolet spectrophotometer, with glucose as the standard curve. One unit of cellulase activity was defined as the amount of enzyme required to produce glucose (1 μg/min) under the above reaction conditions.

During the determination of pectinase activity, 1% CMC-Na solution was replaced by 1% pectin solution, the other methods were similar to those used for cellulase determination. One unit of pectinase activity was defined as the amount of enzyme required to produce glucose (1 μg/min) under the above reaction conditions.

The protease activity was determined using the Folin–Ciocalteu method (Sharma et al., 2021): 1 mL of crude enzyme solution was added to 1 mL of casein. Both the crude enzyme solution and casein substrate were pre-incubated at 40°C in a water bath for 3 min before mixing. Once combined, the reaction mixture was incubated further for 10 min at 40°C in a water bath. Afterward, 2 mL of 0.4 mol/L trichloroacetic acid solution, which is used to inhibit the enzyme, was removed and allowed to sit for 10 min at 20°C and then the solution was centrifuged at 1632 × g for 5 min. After centrifugation, 1 mL of supernatant was added to 5 mL of 0.4 mol/L sodium carbonate solution and then 1 mL of Folin reagent was added to the solution. This was then shaken well and kept in a 40°C water bath for 20 min. An ultraviolet spectrophotometer was used to measure the optical density of the solution at 660 nm, with tyrosine used to plot the standard curve. One unit of protease activity was defined as the amount of enzyme required to hydrolyze casein to produce 1 μg/min tyrosine under the above reaction conditions.

Polyphenol oxidase activity was determined using the catechol method (González-Ceballos et al., 2023): 1.0 mL of crude enzyme solution was added to 3 mL of reaction mixture (citrate buffer: 0.1% proline: 1%, catechol = 10:2:3). The solution was then kept in a thermostatic water bath at 37°C for 10 min, and 3 mL of trichloroacetic acid (1 mL) was added immediately to terminate the reaction. The optical density was measured at 460 nm using an ultraviolet spectrophotometer, and the catechol in the blank control group was replaced by 0.05 mol/L PBS Buffer Solution. One unit of polyphenol oxidase activity was defined as the amount of enzyme required to increase the absorbance by 1 within 1 min under the above reaction conditions.

### 2.4 Data analysis

To ensure the reliability of the test results, all enzymatic activity test experiments were repeated three times. DPS 15.10 (Zheng et al., 2023) statistical software was used to analyze the variance in the data. *p* < 0.05 indicated a significant difference and the test results were expressed as mean ± standard error.

## 3 Results

### 3.1 Morphological diversity and characteristics of different *A. cristatus* strains

**Figure 2:**
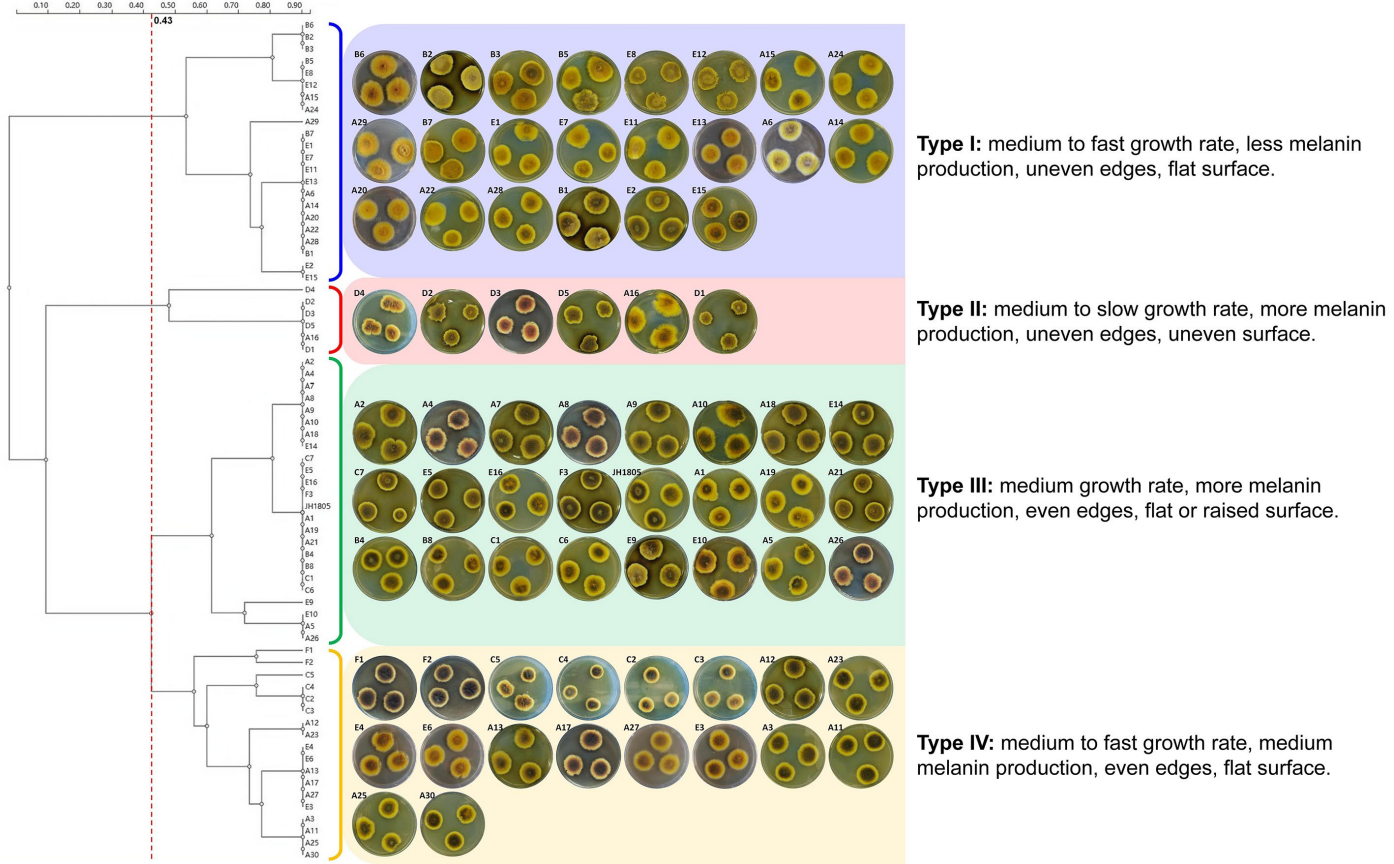
Colony characteristics and morphological clustering of 70 *A. cristatus* cultured in PDA medium (28°C, 6 d).

The 70 *A. cristatus* strains, including the standard JH1805, exhibited several common morphological features (Figure 2). First, the mycelial structure was relatively dense and firmly attached to the medium; colony edges were relatively shallow, mostly light yellow to yellow, while the melanin-dyed central area was orange yellow to brown. The overall colony structure was presented as concentric rings of 2–3 colors. The sporogenous structure was mainly closed ascocarp, and small amounts of gray-green conidia heads were present along the edges of several colonies. Finally, the medium around the colony formed a black-brown halo from pigment diffusion.

The 70 strains varied in growth rate, melanin secretion, colony edge shape, and colony surface characteristics. A significant disparity in growth rate was reflected on day 6, when A2, B1, and E2 colony diameters exceeded 30 mm, whereas A30, D1, and C4 were 18–26 mm in diameter. The A11, A12, and F1 colonies that had high pigment secretion were dyed black-brown to brown, and the dark area accounted for approximately 3/4 of the diameter of the mycelia. In contrast, the dyed area of A1 and C6 were less than half the colony diameter, and the A14, A22, and E7 strains only secreted a small amount of pigment. In terms of colony edge shape, JH1805, A11, E5, and E15 were all relatively uniform, whereas other strains were uneven, as they were either serrated (A9, A12), or petal-shaped (A2, A4). Surface characteristics also differed across strains. Most colonies were flat, with the center only slightly thicker than the edge, although a few strains exhibited notable uplift (A21, C2) or folds (A17, F1). Reverse-side colony color and conidia number also differed between strains.

After investigating growth rate, pigment secretion, colony edge shape, and surface characteristics of the 70 *A. cristatus* cultured on PDA plates (Supplementary Table S5), a clustering analysis was performed (Figure 2). The results indicated that, at a 0.430 threshold, the tested isolates can be divided into four groups.

**Figure 3:**
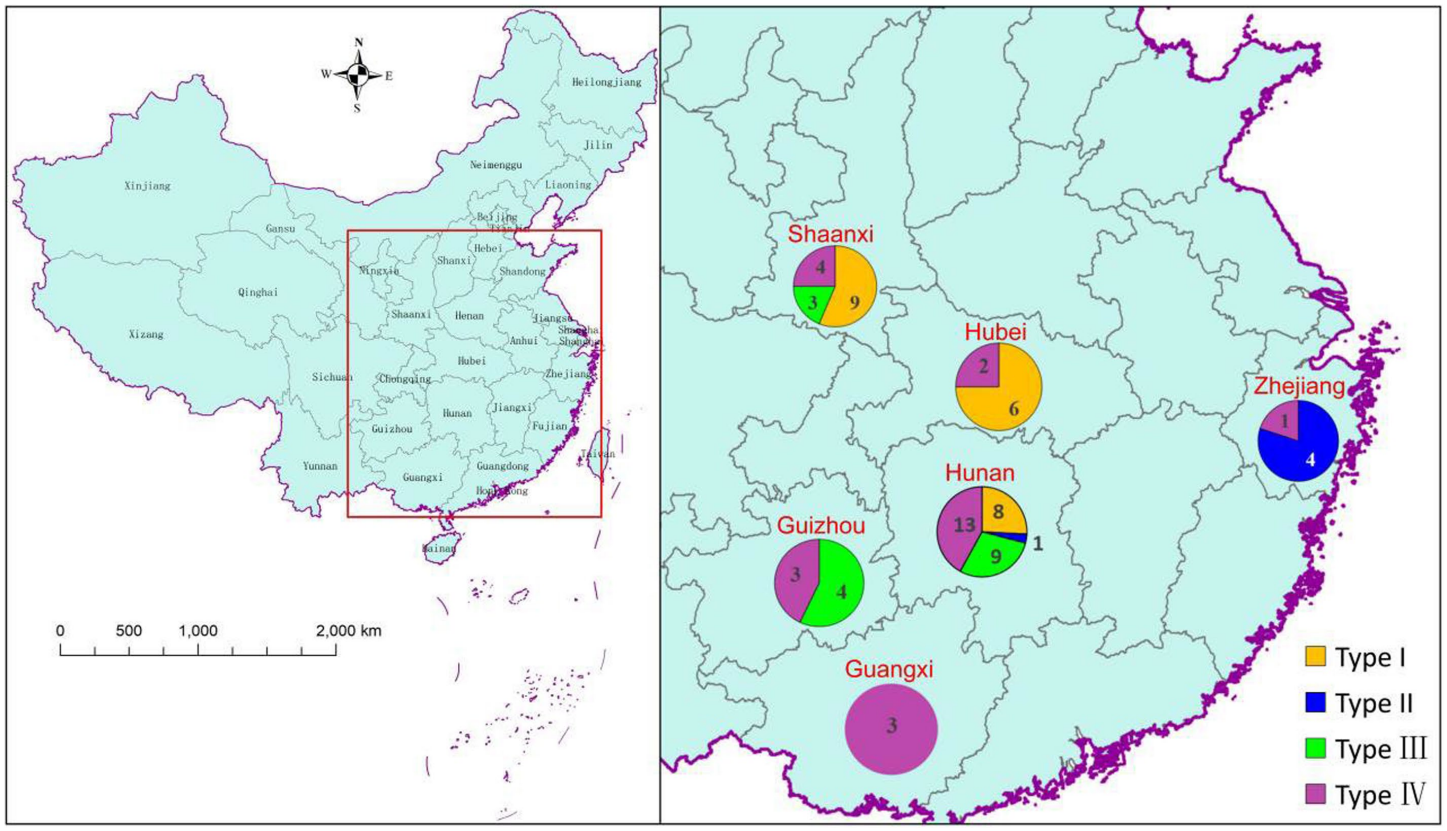
Geographical distribution patterns of various *A. cristatus* types.

To some extent, the tested *A. cristatus* strains were clustered based on geographical distribution (Figure 3). For example, the five *A. cristatus* strains from Zhejiang were clustered in type II, whereas Hubei strains were mainly type I. Type I strains were mainly distributed in the northern dark tea-producing regions, while types III and IV were mainly from the south. Thus, a north–south distribution was observed overall, but no consistent pattern between the distribution type and geographical region was observed.

### 3.2 EST-SSR primer amplification and genetic diversity of *A. cristatus*

The 10,542 transcriptome unigene sequences (totaling 14,972.05 kb in length) yielded 2,183 SSR loci (Table 1). On average, one SSR locus was present for every 6.86 kb sequence. Overall, the SSR frequency was 20.71%, with 358 unigenes containing two or more SSR loci.

**Table 1:** Statistical analysis of SSR loci in the transcriptome of *A. cristatus*.

SSR characteristics	Statistics
Total unigene sequences	10.542
Total length of unigene sequence (kb)	14972.05
Total number of SSR loci	2.183
Number of sequences containing SSR loci	1.680
Number of sequences containing more than one SSR loci	358
Frequency of SSR loci (%)	20.71
Number of composite SSR loci	169

**Figure 4:**
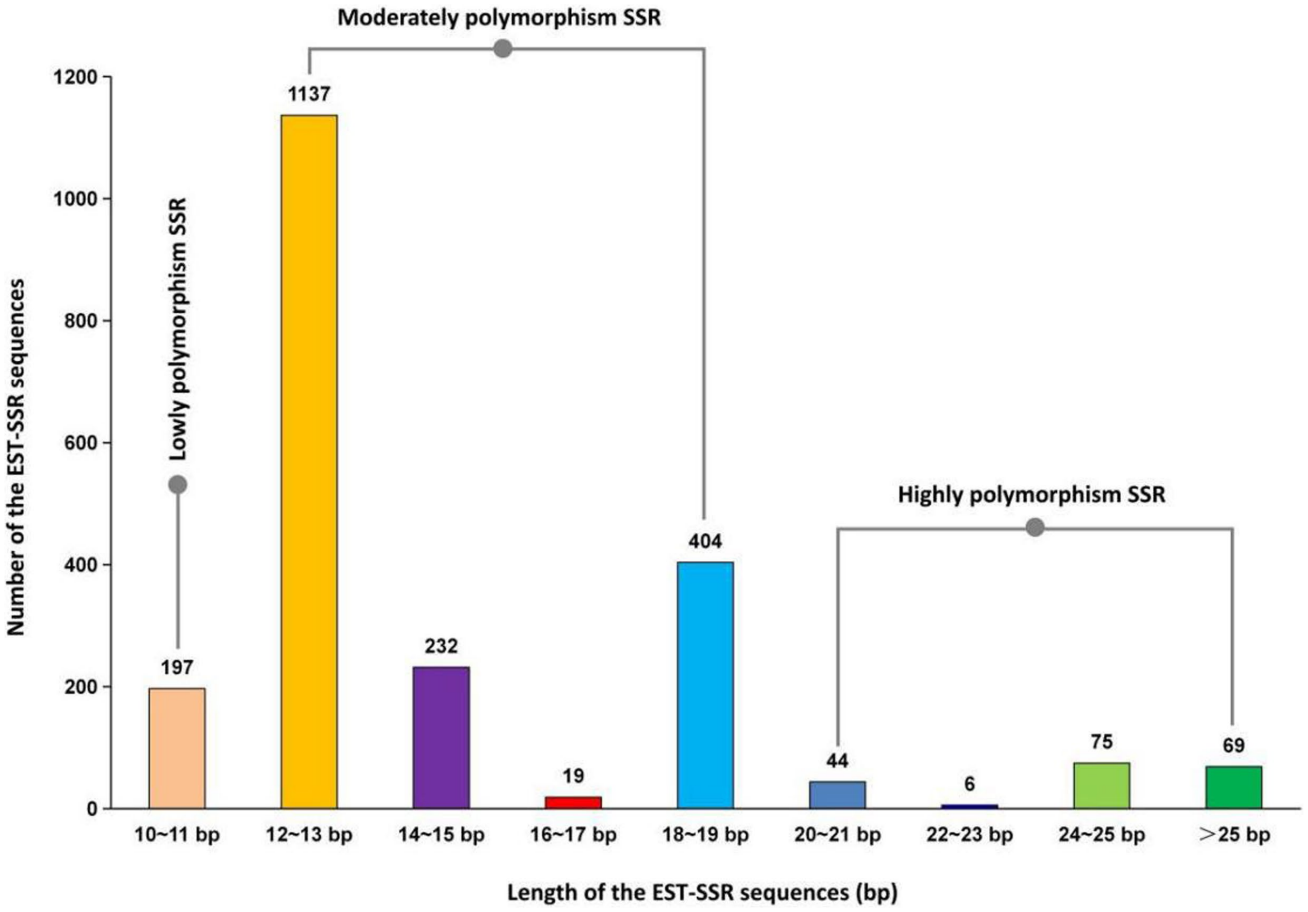
Length distribution of EST-SSR Sequences in *A. cristatus*.

Based on Figure 4, out of 2,183 *A. cristatus* SSR sequences, there are a total of 194 highly polymorphism SSR sequences (8.89%) with a length ≥ 20 bp, 1792 moderately polymorphism SSR sequences (82.09%) with a length between 12–19 bp, and 197 lowly polymorphism SSR sequences (9.02%) with a length < 12 bp. In total, 90.98% of the SSR sequences exhibit moderate to high levels of polymorphism.

Most of the 30 EST-SSR primers successfully amplified the templates from *A. cristatus* (JH1805, A1, A11, A12, A13, A27, A30, B8, C5, C6, D1, E1, E13, B1 and F3) and *A. chevalieri* XW1803 (Supplementary Figure S2).

**Figure 5:**
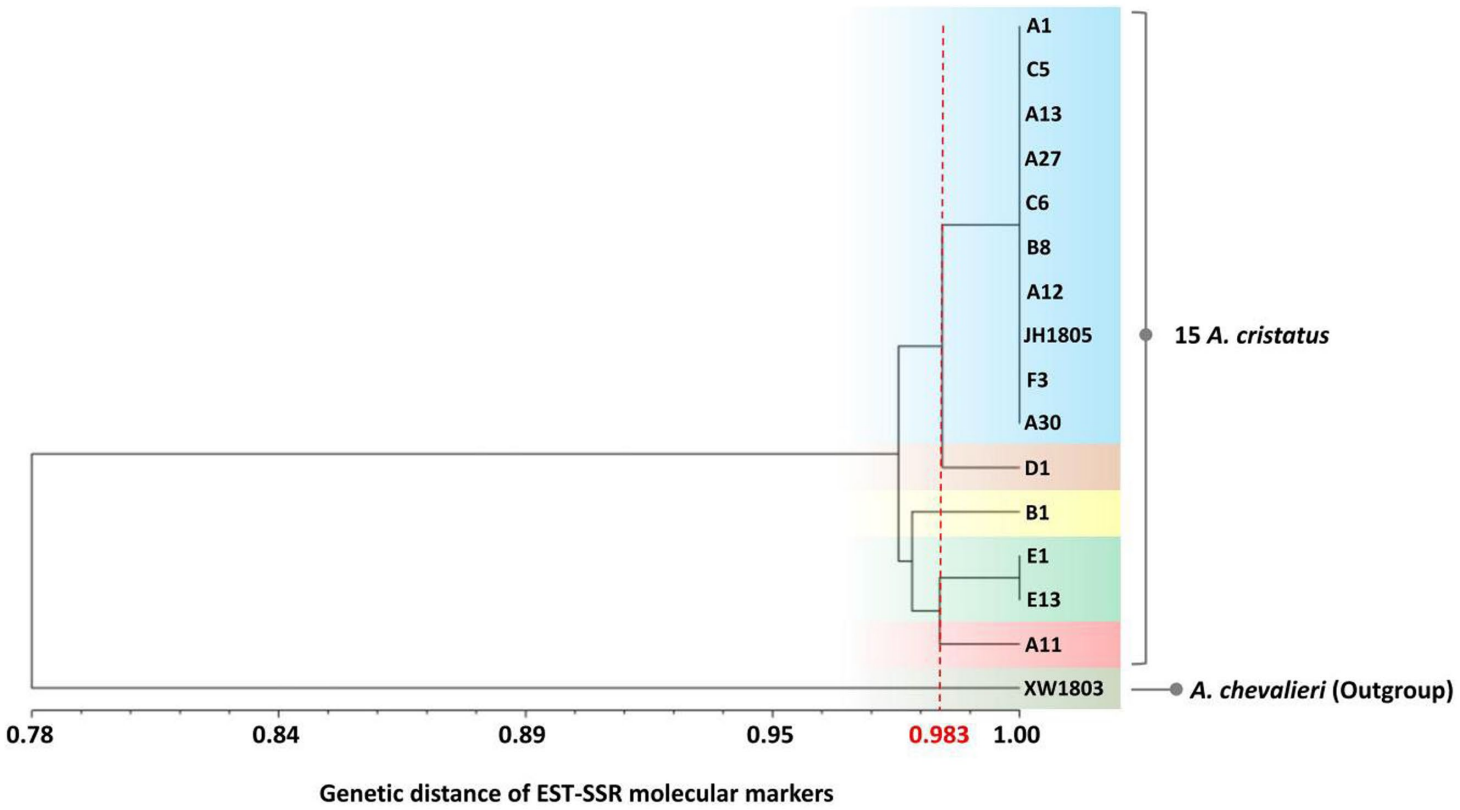
Genetic diversity clustering analysis of 16 fungi was conducted using 30 EST-SSR molecular markers.

Cluster analysis with UPGMA and phylogenetic analyses (Figure 5) revealed that *A. chevalieri* formed a distinct branch, as expected of the outgroup, and exhibited a genetic similarity of 0.775 with *A. cristatus*. The value was significantly lower than the genetic similarity between different *A. cristatus* strains (0.965–0.983). Therefore, the selected primers effectively discriminated between intra- and interspecies units. The 15 *A. cristatus* strains were divided into five branches with a genetic similarity coefficient of 0.983: the first branch included A1, C5, A13, A27, C6, B8, A12, JH1805, F3, and A30; the second branch comprised E1 and E13 from Shaanxi Province; finally, B1, D1 and A11 each formed a branch.

A Mantel correlation analysis was conducted on the Euclidean distance matrix of phenotypic traits and the genetic distance matrix of EST-SSR markers for the 15 *A. cristatus* strains (Supplementary Figure S3). Strain morphology and EST-SSR genetic diversity were significantly correlated (*r* = 0.515, *p* = 0.9959).

### 3.3 Physiological characteristics

#### 3.3.1 Colony characteristics of representative strains

**Figure 6:**
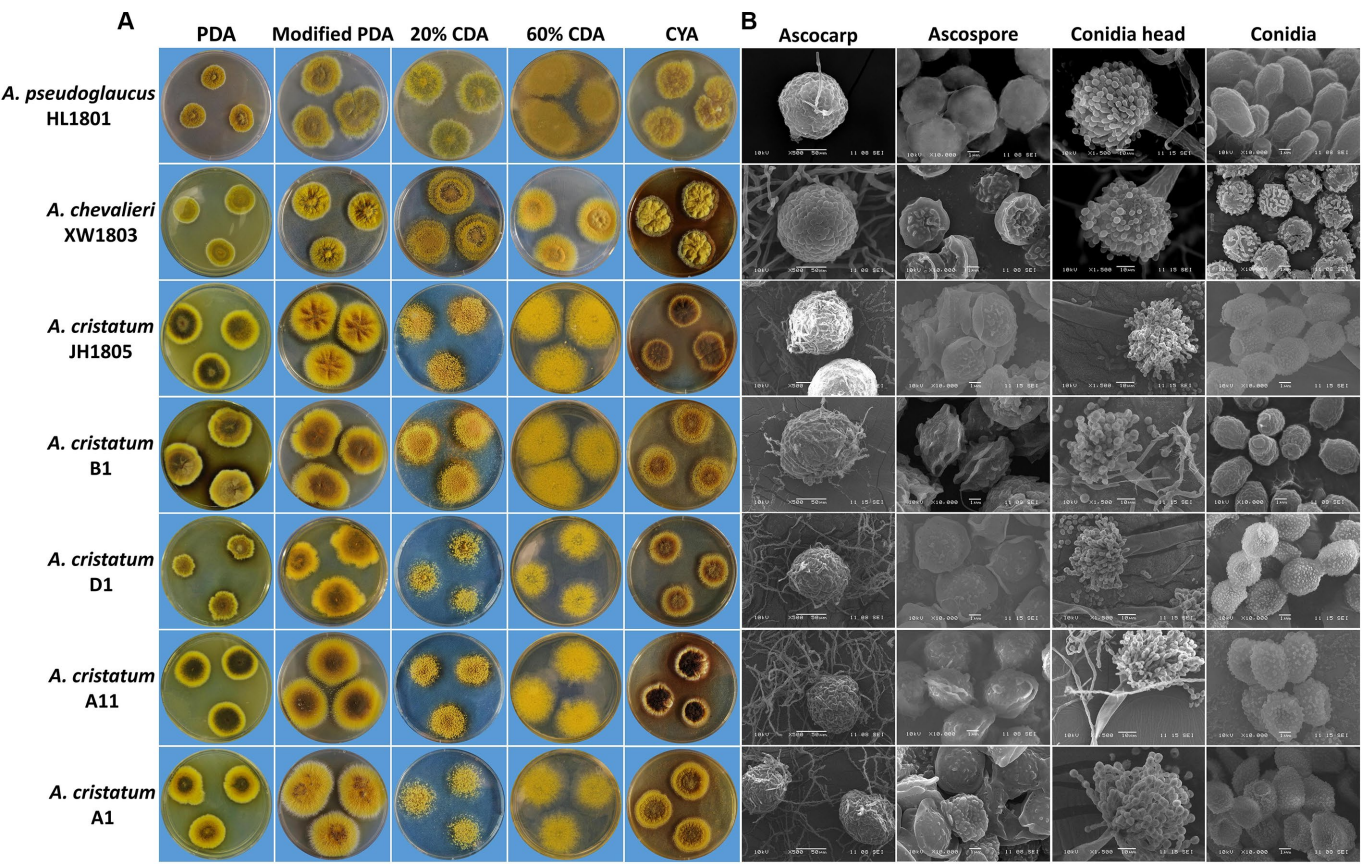
Colony morphology **(A)** and microstructural characteristics under scanning electron microscopy **(B)** of seven *A. cristatus* strains.

All seven tested strains grew successfully on the five cultured media (PDA, modified PDA, 20% CDA, 60% CDA, and CYA), forming colonies with different morphologies (Figure 6A). In general, the growth rate of the seven fungi strains was higher on the modified PDA and 60% CDA media, and lower on the PDA and 20% CDA media, the growth rate of the colony was positively correlated with the concentrations of nutrients, such as carbon source. Moreover, the fungal colonies from the seven strains predominantly displayed ascocarps (structure related to sexual reproduction) grown on the PDA, modified PDA and CYA medium. Conversely, asexual reproduction (conidia head) structures were rarely observed, whereas asexual reproduction was predominantly observed in the colonies grown on the 60% CDA medium. Notably, although the growth rate and pigment secretion of the five strains of *A. cristatus* were affected greatly by the type of medium, the strains showed similar physiological characteristics on different media: for example, B1 grew faster and produced fewer pigments on all the media; A11 grew at a moderate rate and produced more pigments, exhibiting stability of certain physiological characteristics in different strains.

#### 3.3.2 Microscopic characteristics

Strain structural SEM images of seven tested strains were quantified in ImageJ (Figure 6B) and analyzed (Table 2). Sexual *A. cristatus* has a spherical ascocarp (diameter: 50.0–180.0 μm) surrounded by numerous mycelia. As growth progressed, the ascocarp wall became transparent and eventually ruptured, releasing ascospores upon maturation. Ascospores were 3.4–4.7 μm × 4.4–6.4 μm in size and presented a double-convex shape. Their surfaces were rough, with two prominent corona protrusions at the equator and grooves between the protrusions.

**Table 2:** The sizes of different reproductive structures of seven experimental strains.

Strain	Ascocarp (μm)	Ascospore (μm)	Conidia head (μm)	Conidia (μm)
HL1801	70.0–150.0	2.6–4.0 × 4.2–5.6	45.0–70.0	2.7–4.6 × 4.0–7.5
XW1803	65.0–130.0	2.4–3.0 × 5.0–5.4	45.0–65.0	3.2–3.6 × 3.3–4.2
JH1805	55.0–160.0	3.4–4.5 × 4.5–6.1	35.0–55.0	3.3–3.8 × 4.3–5.2
B1	65.0–180.0	3.6–4.7 × 4.6–6.2	40.0–62.0	3.2–3.6 × 4.4–5.0
D1	80.0–175.0	3.7–4.7 × 4.6–6.4	43.0–69.0	3.2–3.5 × 4.3–4.8
A11	50.0–120.0	3.7–4.5 × 4.4–6.0	50.0–80.0	3.4–3.7 × 4.2–4.8
A1	67.0–145.0	3.6–4.6 × 4.5–6.1	48.0–75.0	3.4–3.8 × 4.3–4.9

Asexual *A. cristatus* possessed a conidial head of 35.0–80.0 μm in length. At the immature stage, the conidial head exhibited multiple conidial chains, each containing approximately three to seven conidia, which increased in a time-dependent manner. The mature conidia detached from conidial chains and were dispersed within the matrix. The most mature conidia were observed to be ellipsoidal, with small spiny protrusions on the outer wall and 3.2–3.8 μm × 4.2–5.2 μm in size.

The related *A. pseudoglaucus* is mainly distinguished from *A. cristatus* by a smooth ascospore surface, less obvious coronal protrusions at the equator, and shallower grooves. In *A. chevalieri*, ascospores were flat, with a depression in the middle of the bearing-like equator, while conidia were thicker and shorter.

#### 3.3.3 Extracellular enzyme activity

**Figure 7:**
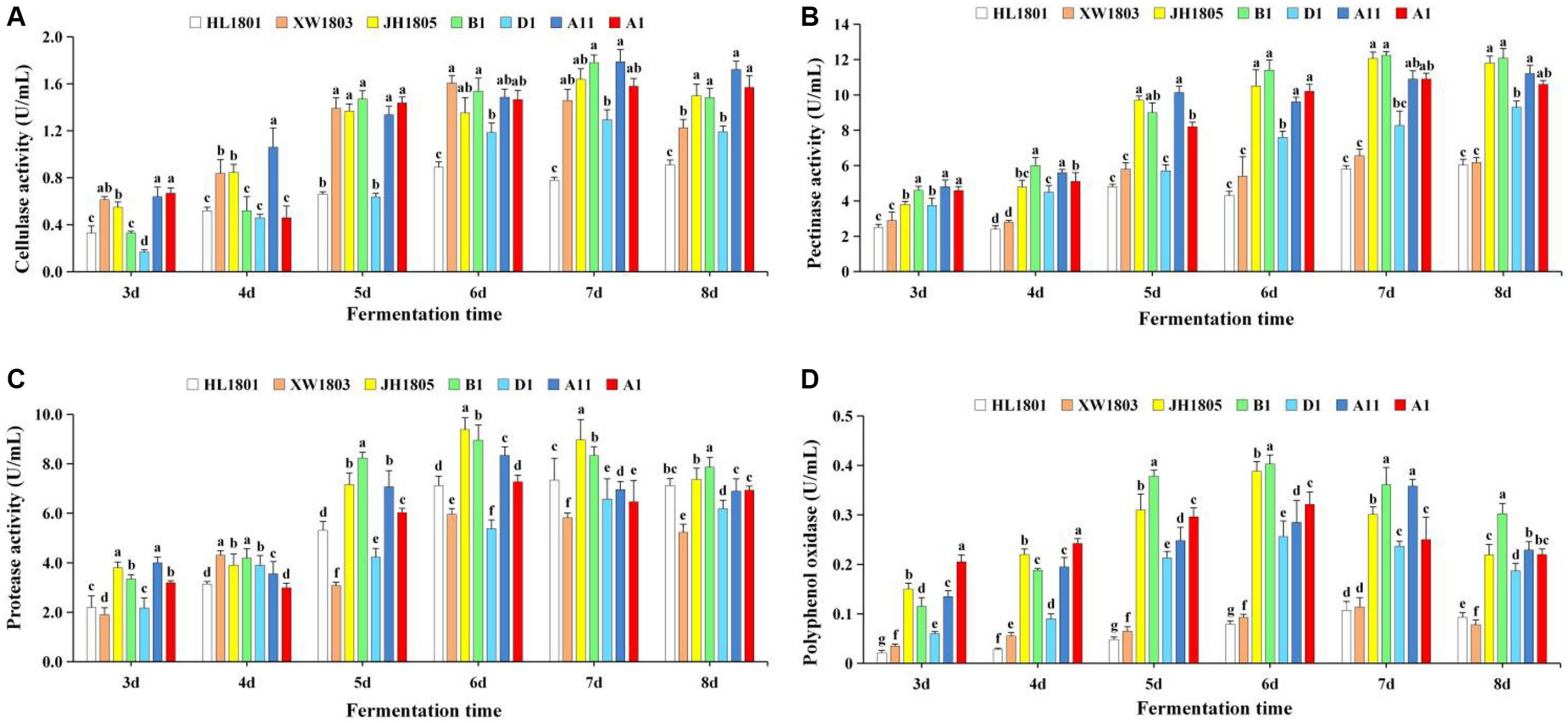
Extracellular enzyme activity in a fermentation broth of seven *A. cristatus* strains [**(A)** Cellulase activity, **(B)** Pectinase activity, **(C)** Protease activity, **(D)** Polyphenol oxidase]. Each column of data for the same fermentation period is marked with a different lowercase letter to indicate significant differences (*p* < 0.05).

In all seven strains, cellulase activity increased rapidly during fermentation, peaking on days 6 and 7 and then leveling off (Figure 7A). Cellulase activity was the highest among *A. cristatus* B1 and A11 (1.779 U/mL and 1.786 U/mL on day 7, respectively). Strong cellulase activity was also observed in *A. chevalieri* XW1803, with a value of 1.607 U/mL on day 6. At the peak of cellulase activity (7 d), the cellulase activity values of each strain were compared in pairs, and there were no significant differences among XW1803, JH1805, B1, A11, and A1 (*p* > 0.05); there were no significant differences among XW1803, JH1805, D1, and A1 (*p* > 0.05); and there were significant differences among the other strains (*p* < 0.05). Among the 5 *A. cristatus*, D1 had the lowest cellulase activity.

The pectinase activity of the seven test strains is shown in Figure 7B. Activity was the highest in JH1805 and B1, peaking on day 7 at 12.071 U/mL and 12.242 U/mL, respectively. In contrast, pectinase activity was low for *A. pseudoglaucus* HL1801 and *A. chevalieri* XW1803, peaking on day 8 (6.029 U/mL) and 7 (6.547 U/mL), respectively. On the 8th day of fermentation, the pectinase activity among the strains was varied. Notably, there were no significant differences observed between strains JH1805, B1, and A11, and A1, as well as between A1 and D1, and between HL1801 and XW1803 (*p* > 0.05). However, the remaining strains exhibited statistically significant differences (*p* < 0.05), demonstrating their diverse methods of hydrolyzing pectin. Notably, most strains maintained high pectinase activity during late fermentation.

Protease activity in all seven strains increased initially before decreasing (Figure 7C). Notably, *A. cristatus* JH1805 and B1 demonstrated robust protease activity, reaching 9.382 U/mL and 8.951 U/mL on day 6, respectively. In contrast, *A. chevalieri* XW1803 and *A. cristatus* D1 had low protease activity, peaking at 5.960 U/mL and 6.568 U/mL on fermentation days 6 and 7, respectively. On the 6th day of the peak period of protease activity, each strain displayed a normal distribution, with significant differences between different strains (*p* < 0.05).

Polyphenol oxidase activity was robust on days 6 and 7 of fermentation, before decreasing (Figure 7D). Activity was particularly high on day 6 in *A. cristatus* B1 and JH1805, peaking at 0.403 U/mL and 0.388 U/mL, respectively. Activity was lower in *A. pseudoglaucus* HL1801 and *A. chevalieri* XW1803, with peak values being 0.107 and 0.114 U/mL, respectively, on day 7. On the 7th day of fermentation, there was no significant difference in polyphenol oxidase activity between B1 and A11, D1 and A1, and HL1801 and XW1803. However, there were significant differences between the other remaining strains (*p* < 0.05).

## 4 Discussion

### 4.1 Morphological classification and distribution characteristics of *A. cristatus*

In the present study, the morphological variations of 70 *A. cristatus* strains (representing six provinces in China) were identified, and they were classified into four distinct groups. A distinct north–south pattern in strain type distribution was observed; however, a full alignment between the distribution and specific geographical regions was absent. The lack of a strong correlation may be attributed to a geographical separation between raw material production and processing for dark tea. For example, in the southern region of Shaanxi Province, the cultivation of *A. cristatus* is limited and the raw materials used for processing dark tea are mainly imported from other provinces (Xiao et al., 2018). Similarly, shortages in locally produced raw tea leaves necessitate the import of these raw leaves from other provinces to maintain the production of processed products. The process of using blended raw materials to produce dark tea is common in the industry (Fu et al., 2016), facilitating the dissemination of *A. cristatus*, and thereby promotes genetic exchange among microbial populations across varying provinces. Additionally, China’s current dark tea production approach is less dependent on artificial fermentation agents; instead, China relies on the natural inoculation of *A. cristatus* to drive the fermentation process (Wu et al., 2023). The use of such a natural method reportedly contributes to the genetic diversity present in the strains involved (Albertin et al., 2014; Steensels et al., 2014).

### 4.2 EST-SSR characteristics and genetic diversity of *A. cristatus*

In the present study, 10,542 Unigenes of *A. cristatus* transcriptome sequences were retrieved, and a total of 2,183 EST-SSR loci were found, with an SSR frequency of 20.71%. Among the EST-SSR sequences in *A. cristatus*, 194 (8.89% of all SSRs) had high polymorphism, and 1,792 (82.09%) were moderately polymorphic. The phylogenetic tree constructed from PCR data showed that *A. chevalieri* clustered separately as an outgroup, with far lower genetic similarity (0.775) than coefficients within *A. cristatus* (0.965–0.983). Furthermore, clustering the strains based on genetic similarity resulted in a strong correlation with morphology-based groups. The results clearly demonstrate that the selected EST-SSR primers effectively identified intra- and inter-species units among the tested strains. Although the clustering tree topology of EST-SSR markers and morphological markers were not completely consistent, the traits of species are usually determined by multiple genes, and the detected EST-SSR loci are not necessarily associated with the traits. The integration of EST-SSR markers and morphological markers enhances the elucidation of the genetic diversity present within a species (Yin et al., 2023b).

### 4.3 Differences in physiological characteristics of representative strains

Morphological differences between *Aspergillus* species (Diba et al., 2007) can be distinguished reliably under different culture media. The results of culturing experiments revealed substantial differences in growth rate, colony shape, and surface color across the five representative *A. cristatus* strains, reflecting physiological differentiation. When compared with the related *A. pseudoglaucus* and *A. chevalieri*, *A. cristatus* morphology was similar to the former on modified PDA and CYA, as well as the latter on modified PDA and 20% CDA. Such similarities often cause confusion of the three fungal species. Changes in media induce morphological differences in *A. cristatus* and related species due to variation in osmotic pressure (Huang et al., 2017; Tan et al., 2018; Hu et al., 2021). In particular, elevated osmotic pressure shifted reproductive mode from sexual to asexual (Ge et al., 2016). The asexual colonies cultured on 60% CDA were different across the three species. Therefore, hypertonic media-induced variation in asexual colonies is an effective means of identifying *A. cristatus* and related species under conventional culture conditions.

Spore characteristics are typically the primary basis for morphological identification of fungi (Simões et al., 2013; van den Brule et al., 2020). Electron microscopy revealed that *A. cristatus* strains had substantially different spores from their relatives. However, ascospores and conidia were highly similar among the five strains, consistent with the findings of previous studies on *A. cristatus* (Ren et al., 2017; Liu et al., 2018; Rui et al., 2019). Slight variations in spore size across the five *A. cristatus* strains (differences in average ascospore and conidia lengths were ≤0.2 μm and ≤0.3 μm, respectively) were observed; however, the fluctuations could be attributed to inconsistencies in maturity. Overall, the microstructure of the five *A. cristatus* strains did not differ considerably.

The extracellular enzyme activity of dominant microorganisms is a key factor promoting fermentation, among which cellulase, pectinase, protease, and polyphenol oxidase are important to the formation of dark tea quality. In tea leaves, cellulase hydrolyzes cellulose to smaller sugar molecules (Bhati et al., 2021), softening leaf stalk tissue and facilitating cell-wall breakdown to enhance water content (Chandini et al., 2011; Xu et al., 2022; Baruah et al., 2023). Pectinase hydrolyzes pectin to generate small water-soluble molecules (de Souza and Kawaguti, 2021), promoting the release of soluble sugars, polysaccharides, and other substances from leaf stalk cells. Such compounds contribute to the flavor profile and clarify tea infusions (Liang et al., 2021; Wang et al., 2023b). Proteases hydrolyze peptide bonds in proteins to improve protein utilization in tea. The generated amino acids contribute components that improve tea aroma and flavor (Yu and Yang, 2020). During tea processing, polyphenol oxidase facilitates the production of pigments, such as theaflavins and thearubigins (Zheng et al., 2016; Wang et al., 2022b; Parveen et al., 2023). This reaction lowers the bitterness of the tea soup, while deepening its color intensity. Furthermore, polyphenol oxidase is critical to tea aroma through the formation of aromatic compounds (e.g., terpenes and vanillin) (Liu et al., 2023b). Notably, the seven strains exhibited distinct activity patterns for all four extracellular enzymes, reflecting variations in metabolic rate and fermentation performance. In addition, the key factor for the formation of dark tea quality is the transformation of its chemical components catalyzed by microbial enzymes. Highly active microbial extracellular enzymes can promote the transformation of its contents, thus shortening the fermentation time and improving the quality of products (Lin et al., 2021). In the present study, three *A. cristatus* strains JH1805, B1, and A11 had stronger extracellular enzyme activities, and they are excellent candidates for use in breeding programs aimed at enhancing dark tea fermentation.

Genetic diversity is the basis of functional diversity and a prerequisite for the development of functional microorganisms. *A. cristatus* is not only a key microorganism for dark tea fermentation, but is also used widely in the development of health products and fungal active substances. However, there is currently less information on the genetic diversity of this strain, which limits the development of its genetic resources and quality control of dark tea. In the present study, a set of EST-SSR markers was developed for the evaluation of genetic resources of *A. cristatus* using high-throughput sequencing technology. The genetic diversity of different *A. cristatus* genetic resources from different sources were analyzed based on EST-SSR markers and phenotypic characteristics, demonstrating the differences in genetic diversity between geographic and temporal locations and providing key molecular marker resources for identification, genetic variation determination, and other *A. cristatus* studies. At the same time, EST-SSR markers originate from transcriptome sequences, reflecting the coding part of genes, and may directly identify the alleles that determine important phenotypes or physiological traits (Yang et al., 2020), providing convenience for *A. cristatus* breeding. Although the application of EST-SSR markers in microorganisms is relatively late, it has the advantages of high polymorphism, good repeatability, codominant inheritance etc. (Zhou et al., 2023), and has great application prospects in the development of *A. cristatus* and other functional microorganisms. Notably, a key limitation of the present study is that only an initial evaluation of the genetic diversity of *A. cristatus* has been completed. Subsequent work can be based on the research results to select core germplasm and further study the physiological characteristics, metabolite activity, and their correlations with molecular markers of the strains, to improve the EST-SSR molecular marker database. In addition, additional *A. cristatus* resources should be collected to enlarge the source range of germplasm resources and facilitate scientific management and exploitation of *A. cristatus* resources.

## 5 Conclusion

This study investigated *A. cristatus* genetic diversity and phylogeny through morphology, microstructure, enzyme activity, and EST-SSR marker analysis. The authors confirmed the presence of distinct populations in *A. cristatus*, identifiable through physiological differentiation and genetic diversity. We further demonstrated the utility of EST-SSR markers as effective tools for strain discrimination. In conclusion, the results have provided a scientific foundation for species identification, genetic diversity analysis, and marker-assisted breeding programs targeting *A. cristatus*.

## Data availability statement

The datasets presented in this study can be found in online repositories. The names of the repository/repositories and accession number(s) can be found below: https://www.ncbi.nlm.nih.gov/genbank/, PRJNA827193.

## Author contributions

ZYH: Writing – original draft, Investigation, Methodology, Writing – review & editing. SQL: Methodology, Writing – review & editing. XHZ: Data curation, Software, Writing – review & editing. ZJL: Data curation, Software, Writing – review & editing. TTL: Investigation, Resources, Writing – review & editing. SLY: Funding acquisition, Writing – review & editing. XYZ: Funding acquisition, Writing – review & editing. ZGX: Methodology, Writing – original draft, Writing – review & editing.

## Supplementary material

SUPPLEMENTARY FIGURE S1 Methods for observation of *A. cristatus* colony morphology. **(A)** Colony size. **(B)** Ability to secrete pigment. **(C)** Colony edge characteristics, and **(D)** Colony surface characteristics.

SUPPLEMENTARY FIGURE S2 Results were obtained from amplification of 30 EST-SSR primers using 16 fungi DNA samples (Lane 1–15: *A. cristatus* A1, B1, C5, D1, A13, A27, C6, E1, A30, F3, JH1805, A12, B8, E13, A11; lane 16: *A. chevalieri* XW1803).

SUPPLEMENTARY FIGURE S3 Correlation of strain morphology with EST-SSR genetic diversity.

SUPPLEMENTARY TABLE S1 Source information of experimental strains.

SUPPLEMENTARY TABLE S2 Different culture medium formulations.

SUPPLEMENTARY TABLE S3 30 EST-SSR primers information of A. cristatus.

SUPPLEMENTARY TABLE S4 Parameter setting for PCR amplification of EST-SSR sequences.

SUPPLEMENTARY TABLE S5 Colony characteristics of 70 A. cristatus on PDA plate (28 °C, 6 d).
