# Supplementary material for: Morphological variation and expressed sequence tags-simple sequence repeats-based genetic diversity of Aspergillus cristatus in Chinese dark tea
Source: Front Microbiol. 2024 Jun 3;15:1390030. doi: 10.3389/fmicb.2024.1390030 (PMC11180798; doi:10.3389/fmicb.2024.1390030)
Supplement: SUPPLEMENTARY FIGURE S1 — Methods for observation of A. cristatus colony morphology. (A) Colony size. (B) Ability to secrete pigment. (C) Colony edge characteristics, and (D) Colony surface characteristics. [file Data_Sheet_1.ZIP › Supplementary Files/Table S1.docx]

**Table S1.** Source information of experimental strains.

| **Strains number** | **Collection location** | **Longitude and latitude** |
| --- | --- | --- |
| JH1805 (Standard strain) | Hunan City University | 112°39′ E, 28°55′ N |
| HL1801 (Standard strain) | Hunan City University | 112°39′ E, 28°55′ N |
| XW1803 (Standard strain) | Hunan City University | 112°39′ E, 28°55′ N |
| A1 | Yiyang City, Hunan Province | 112°36′ E, 28°56′ N |
| A2 | Yiyang City, Hunan Province | 111°48′ E, 28°39′ N |
| A3 | Yiyang City, Hunan Province | 111°28′ E, 28°38′ N |
| A4 | Yueyang City, Hunan Province | 113°50′ E, 29°57′ N |
| A5 | Yiyang City, Hunan Province | 111°27′ E, 28°38′ N |
| A6 | Yueyang City, Hunan Province | 113°62′ E, 29°52′ N |
| A7 | Yiyang City, Hunan Province | 111°39′ E, 28°39′ N |
| A8 | Yiyang City, Hunan Province | 112°35′ E, 28°48′ N |
| A9 | Yiyang City, Hunan Province | 111°53′ E, 28°51′ N |
| A10 | Yueyang City, Hunan Province | 113°42′ E, 29°34′ N |
| A11 | Yiyang City, Hunan Province | 112°41′ E, 28°56′ N |
| A12 | Yiyang City, Hunan Province | 111°77′ E, 28°49′ N |
| A13 | Yueyang City, Hunan Province | 112°83′ E, 28°76′ N |
| A14 | Yiyang City, Hunan Province | 111°24′ E, 28°38′ N |
| A15 | Changsha City, Hunan Province | 113°03′ E, 28°18′ N |
| A16 | Yiyang City, Hunan Province | 111°28′ E, 28°38′ N |
| A17 | Yiyang City, Hunan Province | 111°08′ E, 28°16′ N |
| A18 | Yiyang City, Hunan Province | 111°32′ E, 28°33′ N |
| A19 | Yiyang City, Hunan Province | 112°39′ E, 28°58′ N |
| A20 | Yiyang City, Hunan Province | 111°30′ E, 28°28′ N |
| A21 | Yiyang City, Hunan Province | 111°40′ E, 28°37′ N |
| A22 | Yiyang City, Hunan Province | 111°04′ E, 28°37′ N |
| A23 | Yiyang City, Hunan Province | 113°03′ E, 28°37′ N |
| A24 | Yiyang City, Hunan Province | 111°23′ E, 28°38′ N |
| A25 | Yiyang City, Hunan Province | 111°39′ E, 28°39′ N |
| A26 | Yiyang City, Hunan Province | 111°49′ E, 28°37′ N |
| A27 | Yiyang City, Hunan Province | 111°59′ E, 28°11′ N |
| A28 | Yiyang City, Hunan Province | 111°40′ E, 28°45′ N |
| A29 | Changsha City, Hunan Province | 111°98′ E, 28°22′ N |
| A30 | Yiyang City, Hunan Province | 111°53′ E, 28°51′ N |
| B1 | Xianning City, Hubei Province | 113°73′ E, 29°57′ N |
| B2 | Huanggang City, Hubei Province | 115°69′ E, 30°71′ N |
| B3 | Xiangyang City, Hubei Province | 111°66′ E, 32°27′ N |
| B4 | Xianning City, Hubei Province | 113°72′ E, 29°57′ N |
| B5 | Enshi City, Hubei Province | 109°50′ E, 30°31′ N |
| B6 | Xianning City, Hubei Province | 113°83′ E, 29°69′ N |
| B7 | Enshi City, Hubei Province | 114°31′ E, 30°55′ N |
| B8 | Enshi City, Hubei Province | 109°45′ E, 30°01′ N |
| C1 | Zunyi City, Guizhou Province | 107°50′ E, 27°74′ N |
| C2 | Tongren City, Guizhou Province | 108°33′ E, 27°43′ N |
| C3 | Tongren City, Guizhou Province | 107°93′ E, 27°55′ N |
| C4 | Tongren City, Guizhou Province | 109°19′ E, 28°12′ N |
| C5 | Zunyi City, Guizhou Province | 107°47′ E, 27°75′ N |
| C6 | Tongren City, Guizhou Province | 109°21′ E, 27°74′ N |
| C7 | Zunyi City, Guizhou Province | 107°47′ E, 27°75′ N |
| D1 | Shaoxing City, Zhejiang Province | 120°86′ E, 29°50′ N |
| D2 | Jinhua City, Zhejiang Province | 119°71′ E, 28°83′ N |
| D3 | Shaoxing City, Zhejiang Province | 120°47′ E, 29°98′ N |
| D4 | Jinhua City, Zhejiang Province | 119°71′ E, 28°83′ N |
| D5 | Shaoxing City, Zhejiang Province | 120°61′ E, 29°80′ N |
| E1 | Xianyang City, Shaanxi Province | 108°86′ E, 34°53′ N |
| E2 | Xianyang City, Shaanxi Province | 108°78′ E, 34°32′ N |
| E3 | Xi 'an City, Shaanxi Province | 108°96′ E, 34°26′ N |
| E4 | Xi 'an City, Shaanxi Province | 108°67′ E, 34°21′ N |
| E5 | Xianyang City, Shaanxi Province | 108°85′ E, 34°53′ N |
| E6 | Xianyang City, Shaanxi Province | 108°82′ E, 34°53′ N |
| E7 | Xi 'an City, Shaanxi Province | 108°96′ E, 34°26′ N |
| E8 | Xianyang City, Shaanxi Province | 108°84′ E, 34°53′ N |
| E9 | Xianyang City, Shaanxi Province | 108°80′ E, 34°37′ N |
| E10 | Xianyang City, Shaanxi Province | 108°85′ E, 34°53′ N |
| E11 | Xianyang City, Shaanxi Province | 108°88′ E, 34°54′ N |
| E12 | Xianyang City, Shaanxi Province | 108°85′ E, 34°53′ N |
| E13 | Xianyang City, Shaanxi Province | 108°90′ E, 34°52′ N |
| E14 | Xianyang City, Shaanxi Province | 108°88′ E, 34°54′ N |
| E15 | Xianyang City, Shaanxi Province | 108°91′ E, 34°53′ N |
| E16 | Xianyang City, Shaanxi Province | 108°73′ E, 34°34′ N |
| F1 | Wuzhou City, Guangxi Province | 111°31′ E, 23°51′ N |
| F2 | Wuzhou City, Guangxi Province | 111°33′ E, 23°53′ N |
| F3 | Nanning City, Guangxi Province | 109°28′ E, 22°68′ N |
