# Supplementary material for: Morphological variation and expressed sequence tags-simple sequence repeats-based genetic diversity of Aspergillus cristatus in Chinese dark tea
Source: Front Microbiol. 2024 Jun 3;15:1390030. doi: 10.3389/fmicb.2024.1390030 (PMC11180798; doi:10.3389/fmicb.2024.1390030)
Supplement: SUPPLEMENTARY FIGURE S1 — Methods for observation of A. cristatus colony morphology. (A) Colony size. (B) Ability to secrete pigment. (C) Colony edge characteristics, and (D) Colony surface characteristics. [file Data_Sheet_1.ZIP › Supplementary Files/Table S2.docx]

**Table S2.** Different culture medium formulations.

| **Designation of the culture medium** | **Abbreviation** | **Formulation of culture medium** |
| --- | --- | --- |
| Potato dextrose agar | PDA | Potato 200 g/L, glucose 20 g/L, agar powder 20 g/L |
| Modified potato dextrose agar | Modified PDA | Potato 300 g/L, glucose 80 g/L, NaCl 5 g/L, agar powder 20 g/L |
| 20% Czapek–Dox medium | 20％ CDA | NaNO_3_ 3 g/L, K_2_HPO_4_ 1 g/L, MgSO_4_·7H_2_O 0.5 g/L, KCl 0.5 g/L, FeSO_4_ 0.01 g/L, sucrose 200 g/L, agar powder 20 g/L |
| 60% Czapek–Dox medium | 60％ CDA | NaNO_3_ 3 g/L, K_2_HPO_4_ 1 g/L, MgSO_4_·7H_2_O 0.5 g/L, KCl 0.5 g/L, FeSO_4_ 0.01 g/L, sucrose 600 g/L, agar powder 20 g/L |
| Czapek yeast extract agar | CYA | NaNO_3_ 3 g/L, K_2_HPO_4_ 1 g/L, MgSO_4_·7H_2_O 0.5 g/L, KCl 0.5 g/L, FeSO_4_ 0.01 g/L, sucrose 30 g/L, yeast extract 5 g/L, agar powder 20 g/L |
| Potato glucose liquid medium | PDL | Potato 200 g/L, glucose 20 g/L |
| Dark tea liquid medium | DTL | Raw dark tea 80 g/L, sucrose 50 g/L |
