# Supplementary material for: Morphological variation and expressed sequence tags-simple sequence repeats-based genetic diversity of Aspergillus cristatus in Chinese dark tea
Source: Front Microbiol. 2024 Jun 3;15:1390030. doi: 10.3389/fmicb.2024.1390030 (PMC11180798; doi:10.3389/fmicb.2024.1390030)
Supplement: SUPPLEMENTARY FIGURE S1 — Methods for observation of A. cristatus colony morphology. (A) Colony size. (B) Ability to secrete pigment. (C) Colony edge characteristics, and (D) Colony surface characteristics. [file Data_Sheet_1.ZIP › Supplementary Files/Table S3.docx]

**Table S3.** 30 EST-SSR primers information of *A. cristatus*.

| **Primer name** | **Repeat unit** | **Forward primer** **sequence (5'→3')** | **Reverse primer sequence (5'→3')** |
| --- | --- | --- | --- |
| SSR 1 | C (1×15) | CTATGATTCTCCGGAAGGTGGG | GATTCCCTCGCCGCAGTGT |
| SSR 2 | G (1×23) | TGATTGTCTCCCGTTTATTCCTG | GTGGCTCTTCAACTGTTCTGCTC |
| SSR 3 | A (1×29) | GCAACTGTCGCCTGAGAATGT | AAGAGGTAGCAGTTGTCCCCAC |
| SSR 4 | AGC (3×6) | ACATCCGCCGAAACAACAGA | GCACCGACAGCGATAAAGACA |
| SSR 5 | ACC (3×4) | TCTCCCGAGCGGAATTGAA | CGAAGCGGATGGATTGTTTG |
| SSR 6 | AAAG (4×3) | GGCGAGGCGAGATGTAGAAC | CTGGGCAACAGCATCAATCA |
| SSR 7 | AAG (3×6) | AGCTCAAGTCATCTCGTTCTAGGTT | TTCGCAGAACCAGAGTCCTTG |
| SSR 8 | AAG (3×6) | TCTTCAGATCCTACTTTCCAACCAC | GGTGCTGTTGGTTTAATTCTCTGA |
| SSR 9 | TCG (3×6) | GTGAACATGGGGATATGCGTG | AAGAGGGTGTAGGAGATTTTGGG |
| SSR 10 | CTG (3×7) | GGAAATCGTCAATCATCTCAGCC | TAGAAGGTGGTGTTGCGGGG |
| SSR 11 | AAG (3×7) | CAGACGAGGAAGAGGAAGCGG | GGTTTGGCTGATGTCGTGTGTC |
| SSR 12 | AAG (3×9) | GTCTGCGACTCGGATAGCCA | GTTGTGGGTGTCGCGAATAGAT |
| SSR 13 | AAG (3×7) | GGAAACACCCGTCAAGAGCA | CTTGAAGACGACTCCCAGCATT |
| SSR 14 | AGC (3×11) | ATGGAGCACAATGCCCGG | ACATTGCGGGGTAAACGAGTAA |
| SSR 15 | AGC (3×7) | CACGCCTCGTCCTACTCCG | GGTTGCACCCGCACCAAT |
| SSR 16 | AGC (3×9) | TCTATCTGCGTGGCTGAGGAG | TCAATGACCGATTCACGCTTAG |
| SSR 17 | AGC (3×9) | GAAGATGGCGAAGAGGGTGA | GAGGTTGAGTGGTCTCGGGAG |
| SSR 18 | CCG (3×5) | ACGGATTATCTGCTGTCGTCTTC | ACGTGCTCTTGACGGTAGGAAT |
| SSR 19 | AGAGCC (6×3) | TTGTCACGACCAAGAAGGATAAGA | TCAACAGCCCAAGACCCCA |
| SSR 20 | AGAGCC (6×3) | CAACAAATGGCACCGCTCC | TTGCGCTTCTGCTGTTTGG |
| SSR 21 | ACCAGC (6×3) | AAGCAAGAGCAAGATGAGGAGGA | GGCGGGGTGTGTTTTCTGTAC |
| SSR 22 | AAAAG (5×3) | GATTGCCGGAGAAGGGATG | GTTAGGTAGAATTGCATTAGAGACTGAT |
| SSR 23 | C (1×13) | GCACGAAGAAGGAAAAGGACAGT | CGATGATAGGATGATACTCAGACCG |
| SSR 24 | A (1×16) | TCCGATGGGAGACGAAAACG | GAGAACCAACCAGGAGGCAAA |
| SSR 25 | A (1×23) | ATTTTTCACGAGGCTCAACTTCA | TATTCTAGATGGCCGTTCCGTT |
| SSR 26 | AAG (3×7) | TGAACGAGTGGCTGGAAGATG | GCTGCTGACCCTCCCTATTGT |
| SSR 27 | ACCAGC (6×4) | GAGGAAGAGGAGGTAGAGAACGAC | TGTGTCATGCGGACCGAAA |
| SSR 28 | ATC (3×7) | CCCTAGGCGGAACTATCCATTT | GAAGAGGTTTAGCGTGACGAGTG |
| SSR 29 | AGAGCC (6×4) | TGGTGAGAAAGATACGAACACAGAA | CAGGAGAAGGGACAGCAGGG |
| SSR 30 | AACAGC (6×3) | CGAGGGGCAACAGGTATTCC | TCGGTAGCGTTATCGGTGGT |
