# Supplementary material for: Morphological variation and expressed sequence tags-simple sequence repeats-based genetic diversity of Aspergillus cristatus in Chinese dark tea
Source: Front Microbiol. 2024 Jun 3;15:1390030. doi: 10.3389/fmicb.2024.1390030 (PMC11180798; doi:10.3389/fmicb.2024.1390030)
Supplement: SUPPLEMENTARY FIGURE S1 — Methods for observation of A. cristatus colony morphology. (A) Colony size. (B) Ability to secrete pigment. (C) Colony edge characteristics, and (D) Colony surface characteristics. [file Data_Sheet_1.ZIP › Supplementary Files/Table S4.docx]

**Table S4.** Parameter setting for PCR amplification of EST-SSR sequences.

| **Stages** | **Temperature** | **Time** | **Number of cycles** |
| --- | --- | --- | --- |
| Pre-denaturation | 98 °C | 3 min | 1 |
| Cycle stage | 98 °C | 10 s | 35 |
|  | 59 °C | 10 s |  |
|  | 72 °C | 20 s |  |
| Elongation | 72 °C | 2 min | 1 |
