# Supplementary material for: Morphological variation and expressed sequence tags-simple sequence repeats-based genetic diversity of Aspergillus cristatus in Chinese dark tea
Source: Front Microbiol. 2024 Jun 3;15:1390030. doi: 10.3389/fmicb.2024.1390030 (PMC11180798; doi:10.3389/fmicb.2024.1390030)
Supplement: SUPPLEMENTARY FIGURE S1 — Methods for observation of A. cristatus colony morphology. (A) Colony size. (B) Ability to secrete pigment. (C) Colony edge characteristics, and (D) Colony surface characteristics. [file Data_Sheet_1.ZIP › Supplementary Files/Table S5.docx]

**Table S5.** Colony characteristics of 70 *A. cristatus* on PDA plate (28 °C, 6 d).

| **Strain number** | **Colony size** | **Secretion of melanin** | **Edge of colony** | **Surface of colony** |
| --- | --- | --- | --- | --- |
| JH1805 | 3 | 3 | 1 | 1 |
| A1 | 2 | 2 | 1 | 1 |
| A2 | 3 | 3 | 2 | 1 |
| A3 | 2 | 3 | 1 | 1 |
| A4 | 3 | 3 | 2 | 1 |
| A5 | 2 | 2 | 2 | 1 |
| A6 | 2 | 1 | 1 | 1 |
| A7 | 3 | 3 | 2 | 1 |
| A8 | 3 | 3 | 2 | 1 |
| A9 | 3 | 3 | 2 | 1 |
| A10 | 3 | 3 | 2 | 1 |
| A11 | 2 | 3 | 1 | 1 |
| A12 | 2 | 3 | 2 | 1 |
| A13 | 2 | 3 | 2 | 2 |
| A14 | 2 | 1 | 1 | 1 |
| A15 | 2 | 1 | 2 | 1 |
| A16 | 1 | 2 | 2 | 1 |
| A17 | 2 | 3 | 2 | 2 |
| A18 | 3 | 3 | 2 | 1 |
| A19 | 2 | 2 | 1 | 1 |
| A20 | 3 | 1 | 1 | 1 |
| A21 | 2 | 2 | 1 | 1 |
| A22 | 2 | 1 | 1 | 1 |
| A23 | 2 | 3 | 2 | 1 |
| A24 | 2 | 1 | 2 | 1 |
| A25 | 2 | 3 | 1 | 1 |
| A26 | 2 | 2 | 2 | 1 |
| A27 | 2 | 3 | 2 | 2 |
| A28 | 2 | 1 | 1 | 1 |
| A29 | 3 | 1 | 1 | 2 |
| A30 | 2 | 3 | 1 | 1 |
| B1 | 3 | 2 | 2 | 2 |
| B2 | 3 | 1 | 2 | 1 |
| B3 | 3 | 1 | 2 | 1 |
| B4 | 3 | 3 | 1 | 1 |
| B5 | 2 | 1 | 2 | 1 |
| B6 | 3 | 1 | 2 | 1 |
| B7 | 3 | 1 | 1 | 1 |
| B8 | 2 | 2 | 1 | 1 |
| C1 | 2 | 2 | 1 | 1 |
| C2 | 1 | 3 | 1 | 2 |
| C3 | 1 | 3 | 1 | 2 |
| C4 | 1 | 3 | 1 | 2 |
| C5 | 1 | 3 | 2 | 2 |
| C6 | 2 | 2 | 1 | 1 |
| C7 | 2 | 2 | 1 | 1 |
| D1 | 1 | 2 | 2 | 1 |
| D2 | 1 | 2 | 2 | 1 |
| D3 | 1 | 2 | 2 | 1 |
| D4 | 1 | 2 | 2 | 2 |
| D5 | 1 | 2 | 2 | 1 |
| E1 | 2 | 1 | 1 | 1 |
| E2 | 3 | 2 | 1 | 1 |
| E3 | 2 | 3 | 2 | 2 |
| E4 | 2 | 3 | 2 | 2 |
| E5 | 2 | 2 | 1 | 1 |
| E6 | 2 | 3 | 2 | 2 |
| E7 | 2 | 1 | 1 | 1 |
| E8 | 2 | 1 | 2 | 1 |
| E9 | 3 | 2 | 2 | 1 |
| E10 | 2 | 2 | 2 | 1 |
| E11 | 2 | 1 | 1 | 1 |
| E12 | 2 | 1 | 2 | 1 |
| E13 | 2 | 1 | 1 | 1 |
| E14 | 3 | 3 | 2 | 1 |
| E15 | 3 | 2 | 1 | 1 |
| E16 | 2 | 2 | 1 | 1 |
| F1 | 3 | 3 | 1 | 2 |
| F2 | 2 | 3 | 1 | 2 |
| F3 | 3 | 3 | 1 | 1 |
| **Standard:**  (1) Colony size: 1- Diameter ≤26 mm, 2- Diameter 26-34 mm, 3- Diameter ≥34 mm;  (2) Melanin secretion: 1- A small amount of melanin secretion (melanin area in the center of the colony is not obvious), 2- Moderate level of melanin secretion (a marked melanin area in the center of the colony, but the black circle is light in color; or the black circle is dark in color, but the stained area is small), 3- More melanin secretion (melanin staining area is large and the color is dark);  (3) Colony edge: 1- Tidy edge, 2- Untidy edge;  (4) Colony surface: 1- Flat colony surface, 2- Unflat colony surface (a significant uplift or fold). | | | | |
