# Supplementary figures and images for: Morphological variation and expressed sequence tags-simple sequence repeats-based genetic diversity of Aspergillus cristatus in Chinese dark tea
Source: Front Microbiol. 2024 Jun 3;15:1390030. doi: 10.3389/fmicb.2024.1390030 (PMC11180798; doi:10.3389/fmicb.2024.1390030)

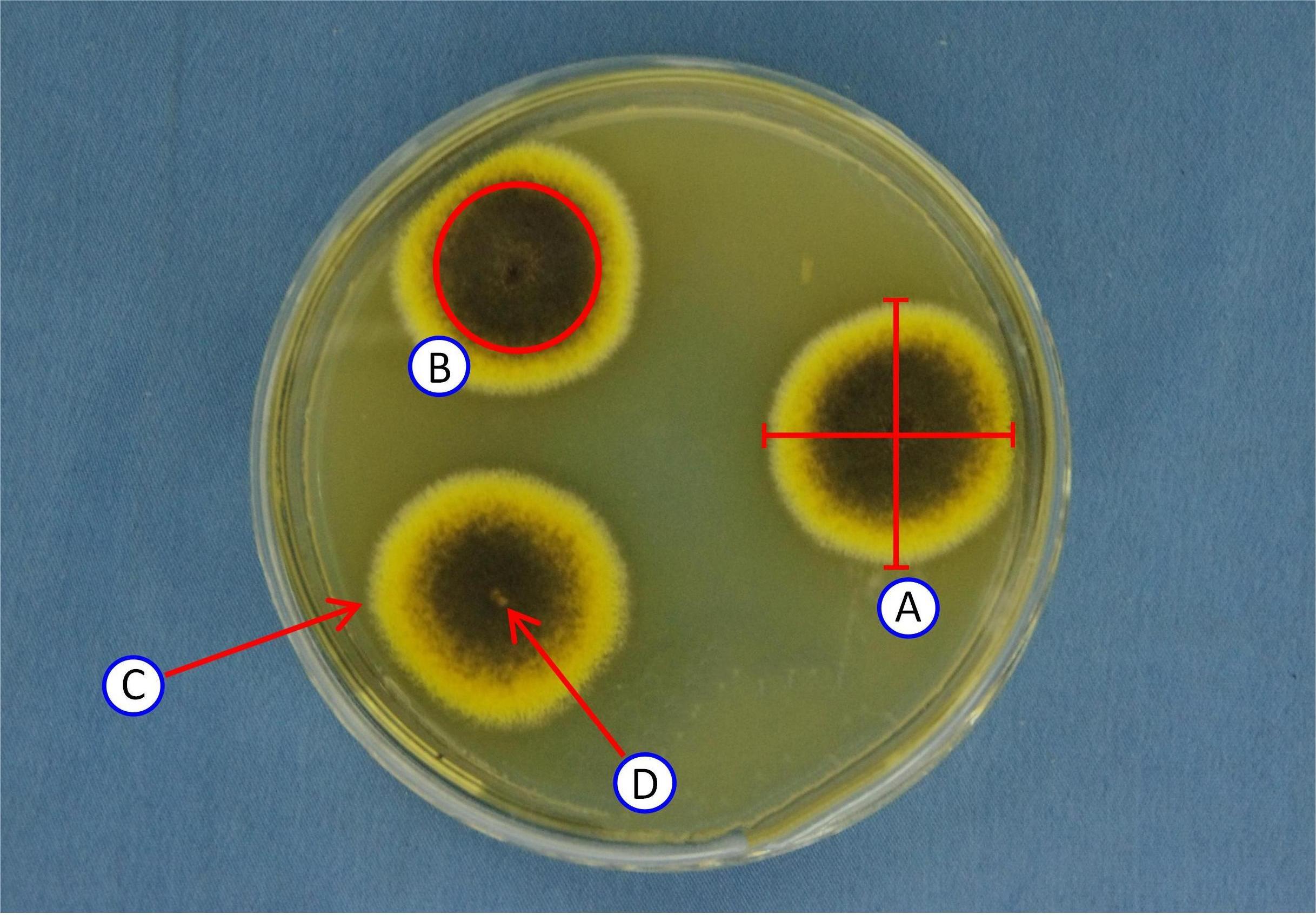

Supplement: SUPPLEMENTARY FIGURE S1 — Methods for observation of A. cristatus colony morphology. (A) Colony size. (B) Ability to secrete pigment. (C) Colony edge characteristics, and (D) Colony surface characteristics. [file Data_Sheet_1.ZIP › Supplementary Files/Figure S1.jpg]

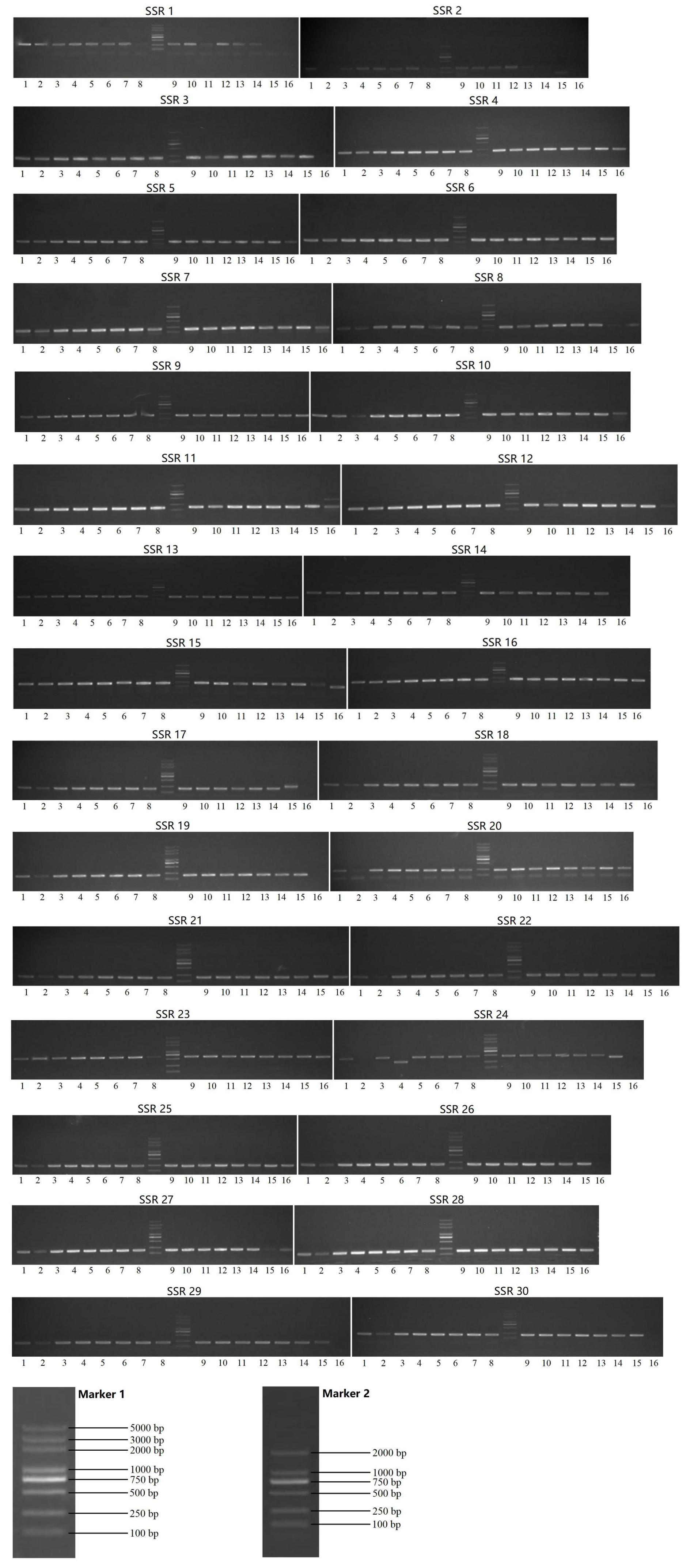

Supplement: SUPPLEMENTARY FIGURE S1 — Methods for observation of A. cristatus colony morphology. (A) Colony size. (B) Ability to secrete pigment. (C) Colony edge characteristics, and (D) Colony surface characteristics. [file Data_Sheet_1.ZIP › Supplementary Files/Figure S2.jpg]

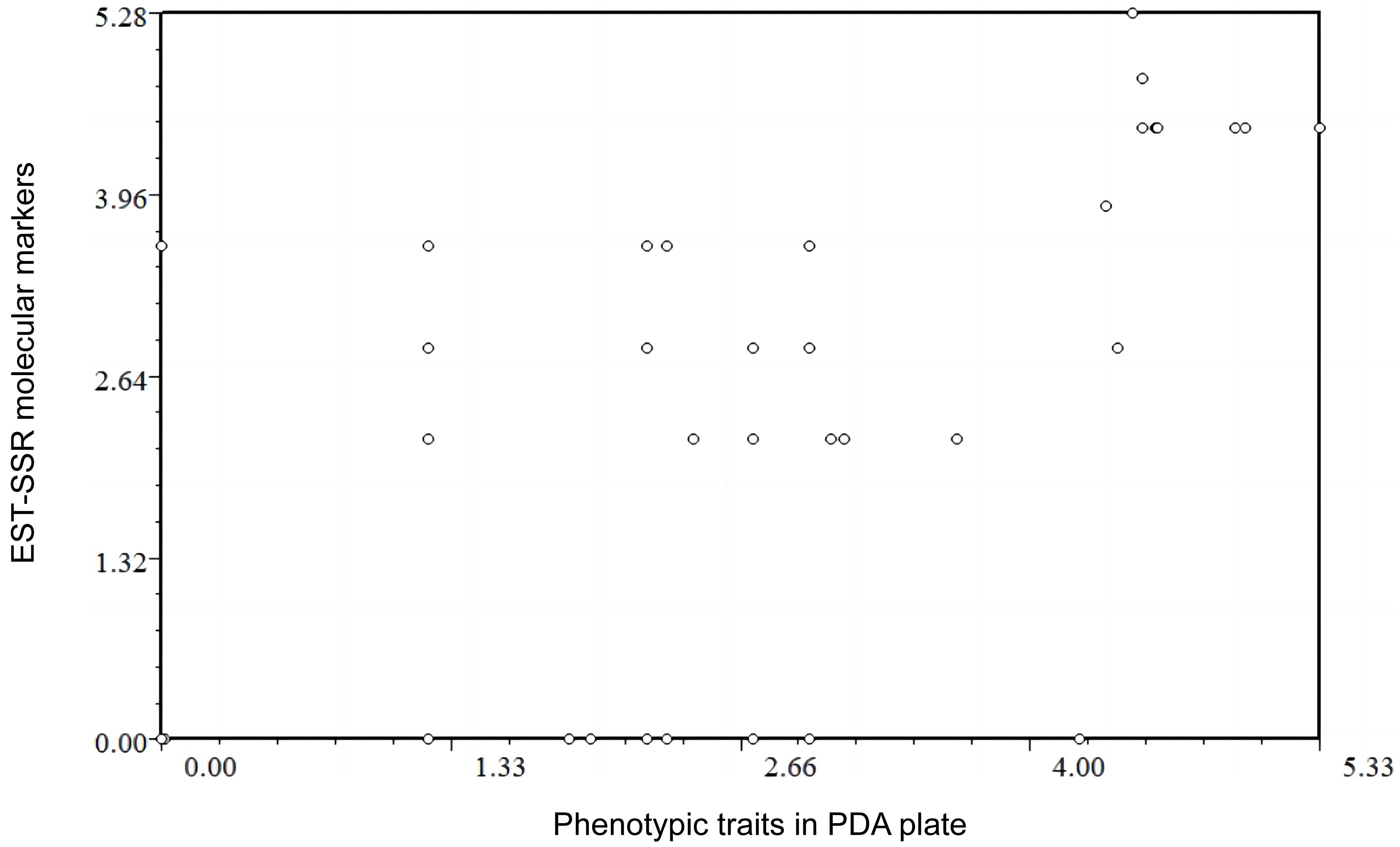

Supplement: SUPPLEMENTARY FIGURE S1 — Methods for observation of A. cristatus colony morphology. (A) Colony size. (B) Ability to secrete pigment. (C) Colony edge characteristics, and (D) Colony surface characteristics. [file Data_Sheet_1.ZIP › Supplementary Files/Figure S3.jpg]
